# Treatment of aggressive T-cell lymphoma/leukemia with anti-CD4 CAR T cells

**DOI:** 10.3389/fimmu.2022.997482

**Published:** 2022-09-12

**Authors:** Jia Feng, Haichan Xu, Andrew Cinquina, Zehua Wu, Wenli Zhang, Lihua Sun, Qi Chen, Lei Tian, Le Song, Kevin G. Pinz, Masayuki Wada, Xun Jiang, William M. Hanes, Yupo Ma, Hongyu Zhang

**Affiliations:** ^1^ Department of Hematology, Peking University Shenzhen Hospital, Shenzhen, China; ^2^ iCell Gene Therapeutics LLC, Research & Development Division, Long Island High Technology Incubator, Stony Brook, NY, United States; ^3^ Department of Hematology, Peking University Third Hospital, Beijing, China; ^4^ Department of Nuclear Medicine, Peking University Third Hospital, Beijing, China

**Keywords:** T-cell lymphoma, hematopoietic cells, CAR T cells, CD4 CAR, IL15/IL15sushi

## Abstract

T-cell lymphomas are aggressive lymphomas that often resist current therapy options or present with relapsed disease, making the development of more effective treatment regimens clinically important. Previously, we have shown that CD4 CAR can effectively target T-cell malignancies in preclinical studies. As IL-15 has been shown to strengthen the anti-tumor response, we have modified CD4 CAR to secrete an IL-15/IL-15sushi complex. These CD4-IL15/IL15sushi CAR T cells and NK92 cells efficiently eliminated CD4+ leukemic cell lines in co-culture assays. Additionally, CD4-IL15/IL15sushi CAR out-performed CD4 CAR in *in vivo* models, demonstrating a benefit to IL-15/IL-15sushi inclusion. In a Phase I clinical trial, CD4-IL15/IL15sushi CAR T cells were tested for safety in three patients with different T-cell lymphomas. Infusion of CD4-IL15/IL15sushi CAR T cells was well-tolerated by the patients without significant adverse effects and led to the remission of their lymphomas. Additionally, infusion led to the depletion of CD4+ Treg cells and expansion of CD3+CD8+ T cells and NK cells. These results suggest that CD4-IL15/IL15sushi CAR T cells may be a safe and effective treatment for patients with relapsed or refractory T-cell lymphomas, where new treatment options are needed.

## Introduction

T-cell lymphomas comprise approximately 10-15% of all non-Hodgkin’s lymphomas (NHL) (1, 2). These lymphomas are further divided into two main subsets: peripheral T-cell lymphomas (PTCLs) and cutaneous T-cell lymphomas (CTCLs). Whereas PTCLs refer to nodal or systemic lymphomas, CTCL refers to lymphomas that originate within the skin. While T-cell lymphomas comprise a small fraction of NHLs, they carry a poorer prognosis with fewer treatment options compared to B-cell lymphomas (3, 4). Standard treatment options for PTCL include cyclophosphamide, doxorubicin, vincristine, and prednisone (CHOP) or CHOP-like therapies. However, therapy remains unsatisfactory, with short survival times for patients with refractory or relapsed disease (5, 6). While early CTCL often have excellent prognosis, advanced or relapsed disease has more limited treatment options and poor outcomes (7).

Chimeric antigen receptor (CAR) T-cell immunotherapy has been extensively reported for B-cell malignancies, such as B-cell acute lymphoblastic leukemia (B-ALL), with high rates of complete remission (8, 9). However, utilization against T-cell malignancies has been more limited. Potential targets, such as CD7 and CD5, are also expressed on T cells undergoing CAR transduction, leading to a significant risk of fratricide. However, both CD7 CAR and CD5 CAR T cells have been successfully developed recently. CD7 CAR T cells that have been genetically modified to prevent surface CD7 expression have demonstrated high rates of remission in patients with relapsed/refractory T-cell acute lymphoblastic leukemia (T-ALL) (10, 11). Similarly, CD5 CAR T cells have been shown to decrease surface expression of CD5 themselves, leading to favorable outcomes in patients with T-ALL (12–14).

PTCLs and CTCLs also commonly have high and persistent expression of CD4, making it another potential target for treatment. Additionally, CD4 is not expressed in hematopoietic stem cells or in non-hematological tissue, minimizing the risk of off-target effects. CD4 has been a widespread target in clinical trials involving monoclonal antibodies for autoimmune disorders, as well as PTCLs and CTCLs (15–21). Overall, short-term CD4 depletion is well-tolerated, reversible, and often no significant clinical evidence of immunosuppression is detected (18). However, targeting CD4 with antibodies has met only limited success in T-cell malignancies, suggesting that alternative and more potent therapies targeting CD4 are required (18, 22). Previously, we have demonstrated that CD4 CAR T cells have potent effects against T-cell malignancies *in vitro* and *in vivo* (23, 24). Therefore, the use of CD4 CAR T cells may have more success in treating T-cell malignancies than an antibody-based approach.

Additionally, the use of CD4 CAR T cells may be beneficial through targeting regulatory T (Treg) cells. Treg cells are CD4+ T cells important for peripheral tolerance to prevent autoimmune-mediated tissue damage through the suppression of effector immune cells. However, Treg can have negative effects on cancer prognosis by decreasing the immune response, including the response by CAR T cells (25–27). By reducing levels of Treg cells, CD4 CARs may avoid this immunosuppression, which could lead to more profound CAR T cell expansion and anti-tumor activity.

As cytokines have potent effects on lymphoid growth, differentiation, and function, they may have a substantial role in the success of immunotherapy outcomes. IL-2 and IL-15 have both been studied extensively for their role in enhancing lymphocyte response and for their potential in the treatment of various malignancies. IL-2 is associated with significant expansion of naïve T-cell populations into effector populations and with activation-induced cell death (AICD) (28, 29). In contrast, IL-15 promotes memory subtypes of T cells and inhibits AICD, leading to increased lymphocyte persistence (28, 29). As persistence of engraftment for at least several months and memory CAR T cell phenotype is likely needed for optimal outcomes, IL-15 is a promising modulator for CAR T cell immunotherapy (30, 31).

Tethered and secreted IL-15 have been reported in studies of CAR against both hematologic and solid malignancies, with increased CAR persistence and improved *in vivo* survival (32–34). While IL-15 alone has a half-life of only ~1 hour, linking it to its soluble receptor, IL-15Rα, has extended its half-life to ~20 hours (35). This IL-15/IL-15Rα complex resulted in increased survival of memory T cells and NK cell, leading to improved outcomes in mouse models of cancer (35, 36). Both IL-15 and IL-15/IL-15Rα have been utilized in clinical trials, with improvements in relapsed or refractory malignancy (37–40). We have also recently shown that CD5 CAR T cells that secrete an IL-15/IL-15Rα compound is safe and led to the remission of a patient with T-cell lymphoblastic leukemia (T-LBL) with CNS infiltration (14).

To target CD4+ PTCLs and CTCLs, we modified CD4 CAR, which was previously used in preclinical studies, to secrete a soluble IL-15 protein linked to the IL-15Rα sushi domain of the IL-15 receptor (abv. CD4-IL15/IL15sushi CAR). This novel CD4-IL15/IL15sushi CAR then demonstrated potent *in vitro* and *in vivo* anti-tumor efficacy in multiple CD4+ cell lines, confirming that the addition of the IL15/IL15sushi complex enhanced anti-tumor targeting and killing. Given these favorable results, CD4-IL15/IL15sushi CAR T cells were then tested for safety in patients with T-cell malignancies in the initial stages of a pilot phase I dose escalation clinical trial (NCT04162340). The results of the first three patients enrolled in the trial are reported here. In addition to safety, the effects of CD4-IL15/IL15sushi CAR T cell on disease remission as well as immune milieu were investigated, to explore the potential of CD4-IL15/IL15sushi CAR T cells in treating T-cell malignancies. No severe adverse effects or opportunistic infections were observed in any patients, and there was transient Grade I (1/3) and Grade II (2/3) cytokine release syndrome (CRS). Infusion of CD4-IL15/IL15sushi CAR T cells led to the rapid decline of CD4+ T cells, leading to the remission of their lymphomas. Remarkably, CD3+CD8+ T cells and NK cells underwent marked expansion in the first month post-infusion, despite the loss of CD4+ T cells. Additionally, immunosuppressive Treg levels were strongly suppressed by CD4-IL15/IL15sushi CAR T cells in the first month post-infusion. Each patient remains in remission of their lymphomas for at least 5 months.

## Materials and methods

### Blood donors and cell lines

Normal peripheral blood mononuclear cells (PBMCs) were obtained from healthy donors’ residual samples using a protocol approved by the Institutional Review Board of Stony Brook University. NK92 cell line was obtained from ATCC (Manassas, VA, USA), and cultured in filtered media, defined as MEM-alpha, 10% FBS, 1% Pen/Strep and supplemented with IL-2 (300 IU/mL; Peprotech, Rocky Hill, NJ, USA), unless otherwise specified. T cells were cultured in filtered T-cell media, defined as 50% AIM V, 40% RPMI 1640 and 10% FBS, with 1% Pen/Strep (all Gibco, Waltham, MA, USA) and supplemented with IL-2 (300 IU/mL), unless otherwise specified. MOLM13, HL60, Jurkat, Karpas299 and MOLT4 cell lines were obtained from ATCC (Manassas, VA, USA), and were cultured in RPMI, 10% FBS, 1% Pen/Strep (Gibco), unless otherwise specified.

For the *in vivo* studies, MOLM13 and Jurkat cell lines were transduced with lentiviral vector expressing luciferase and sorted by resistance to 50 mg/mL puromycin (Sigma Aldrich, St. Louis, MO).

### Production of lentiviral vector

HEK293T cells were cultured in T flasks until 70-80% confluence was reached. Cells were then transfected with the expression plasmid containing CD4-IL15/IL15sushi CAR, and viral packaging plasmids, using the calcium phosphate method (CaCl_2_ solution, 2xHBS). Cells were incubated with transfection solution in DMEM supplemented with 2% FBS for 6-8 hours, when it was removed and replaced with DMEM with 10% FBS, 50 mM HEPES (Gibco), 1x sodium pyrophosphate (Gibco), sodium butyrate (Millipore, 1 mM), and Pen/Strep. After 24 hours incubation, this supernatant was harvested, and filtered through a 0.2 uM disc filter, and stored short-term at 4°C or long-term at -80°C.

### Characterization of CAR T cells

PMBC buffy coat was activated for 48 hours in the presence of 50 ug/mL anti-human CD3 antibody (Miltenyi) in AIM V culture media supplemented with 10% FBS, Pen/Strep, and 300 IU/mL IL-2. Activated T cells were washed, then transduced with either CD4 CAR (*in vivo* experiments only) or CD4-IL15/IL15sushi CAR lentiviral supernatant. 48 hours after transduction, cells were harvested, washed, and moved to tissue culture plates with fresh media and IL-2, as above. After 2 days incubation, cells were harvested and stained first with goat-anti-mouse F(Ab’)_2_, (Jackson Immunoresearch, West Grove, PA). Cells were then washed and stained with streptavidin-PE conjugate (Jackson) and mouse anti-human CD3 and CD4 (Tonbo Biosciences, San Diego, CA), washed, suspended in 2% formalin, and analyzed by flow cytometry (FACSCalibur, BD).

### Characterization of CAR NK92 cells

Cultured NK92 cells were transduced with CD4-IL15/IL15sushi CAR lentiviral supernatant. 48 hours after transduction, cells were harvested, washed, and moved to tissue culture plates with fresh media and IL-2, as above. After 2 days incubation, cells were harvested and stained first with goat-anti-mouse F(Ab’)_2_, (Jackson Immunoresearch, West Grove, PA). Cells were then washed and stained with streptavidin-PE conjugate (Jackson) and mouse anti-human CD56 (Tonbo Biosciences, San Diego, CA), washed, suspended in 2% formalin, and analyzed by flow cytometry (FACSCalibur, BD) to determine transduction efficiency. Expanded cells were subsequently sorted, using the same labeling method, to achieve >95% CAR+ phenotype using FACSAria (BD Biosciences).

### Co-culture target cell ablation assays

In the CAR T cell co-cultures, either CD4-IL15/IL15sushi CAR T cells or blank vector T cells (control) were incubated with target cells at ratio 2:1 (contained 200,000 effector cells to 100,000 target cells) in 1 mL T cell culture media without IL-2 for 24h. Target cells were wild-type MOLM13, HL60, Jurkat, or MOLT4 cells. Each cell line was pre-labeled with CellTracker (CMTMR; Invitrogen) to help distinguish them from T cells. Following co-culture, cells were labeled and analyzed by flow cytometry using mouse anti-human C4 (Tonbo Biosciences, San Diego, CA) antibodies.

In the CAR NK92 cell co-cultures, either CD4-IL15/IL15sushi CAR cells or blank vector NK92 cells (control) were incubated with target cells at ratios of 1:1 (MOLT4) or 5:1 (Karpas) in 1 mL T cell culture media without IL-2 for 24h. Target cells were either wild-type Karpas299 or MOLT4 cells. Following co-culture, cells were labeled and analyzed by flow cytometry using mouse anti-human C4 and CD56 (Tonbo Biosciences, San Diego, CA) antibodies.

All of the co-culture assays were performed in at least two independent experiments. Analysis of anti-leukemic activity was performed by comparing the residual number of cells left in the CD4-IL15/IL15sushi CAR T or NK92 treated samples with the control cells treated samples, and data was presented as the tumor lysis percentage. Analysis was performed using Kaluza software (Beckman Coulter, Brea, CA, USA).

### *In vivo* mouse xenogenic model

NSG mice (NOD.Cg-*Prkdc^scid^ Il2rg^tm1Wjl^*/SzJ) from the Jackson Laboratory (Bar Harbor, ME, USA) were used under a Stony Brook University IACUC-approved protocol. Mice were all male and between 9 and 12 weeks old.

For the *in vivo* experiments using CAR T cells, NSG mice were sub-lethally gamma irradiated (2.0 Gy) and 24 hours later intravenously injected *via* tail vein with 1.0x10^6^ luciferase-expressing MOLM13 cells, an acute myeloid leukemia cell line that is 100% CD4+ to induce measurable tumor formation. Three days following tumor cell injection, 6 mice were intravenously injected *via* tail vein with a course of 8x10^6^ vector control, CD4 CAR, or CD4-IL15/IL15sushi CAR T cells. On days 3, 6, 9, and 11, mice were injected subcutaneously with RediJect D-luciferin (Perkin Elmer, Waltham, MA) and subjected to IVIS imaging (Perkin Elmer) to measure tumor burden.

For the *in vivo* experiments using CAR NK92 cells, to create a stressful condition, we utilized CAR transduced into NK92 cells and Jurkat tumor cells. NK92 cells bear a short half-life, and Jurkat cells had lower CD4 expression. NSG mice were sub-lethally irradiated and intravenously injected with 1.0x10^6^ luciferase-expressing Jurkat cells to induce measurable tumor formation (as above). Three days following tumor cell injection, 5 mice were intravenously injected with a course of 10x10^6^ vector control, CD4 CAR, or CD4-IL15/IL15sushi CAR NK92 cells. On days 3, 7, 10, and 14, mice were injected subcutaneously with RediJect D-luciferin and subjected to IVIS imaging to measure tumor burden.

Images were obtained using Caliper Life Sciences software (PerkinElmer), and data was analyzed to determine tumor burden of treated mice relative to control mice.

### Statistics analysis

The data are presented as the mean ± SEM. The results were analyzed by unpaired Student’s t-test (two-tailed) with Bonferroni correction for multiple comparisons, where applicable. Survival curves were analyzed with the log-rank test. Statistical significance was defined as P < 0.05. All statistical analyses were performed with Prism software version 6.0 (GraphPad).

### Patients and trial design

This is a single-center phase I study of patient-derived CD4-IL15/IL15sushi CAR T-cell therapy for patients with PTCL/CTCL (NCT04162340). Patients were required to provide informed written consent. Eligible patients included patients aged 18 years or older with a CD4+ T-cell lymphoma/leukemia that has not responded to standard therapeutic options (patients who have undergone prior transplant and patients with an inadequate response after 4-6 cycles of standard chemotherapy). Systemic usage of immunosuppressive drug/corticosteroid must have been stopped for more than 1 week prior to treatment. Other criteria included creatinine < 2.5 mg/dL, ALT/AST < 3x upper limit normal, and bilirubin < 2.0 mg/dL. Adequate venous access for apheresis and no contraindication for leukapheresis were required. Patients were excluded if there was a prior solid organ transplant, potentially curative therapy available, uncontrolled active infection, previous treatment with any gene therapy products, if pregnant/lactating, or if any uncontrolled active medical disorder that would preclude participation was present.

CAR T cells were manufactured in a Good Manufacturing Practice laboratory (GMP-lab). Peripheral blood apheresis PBMCs were obtained from the patients. PBMCs were then isolated by Ficoll density gradient centrifugation. Pan T cells were activated by incubation with mouse anti-human CD3 antibody (Miltenyi) for 48 hours. Activated T cells were transduced with lentiviral vector CD4-IL15/IL15sushi for 48 hours. After lentiviral transduction, CAR cells were cultured in AIM V/RPMI medium, containing human serum, and 300 IU/mL IL-2 until day 10. Before infusion, the CAR T cells were subjected to detection of pathogenic microorganisms and contaminants (for example, bacteria, fungi, virus, mycoplasma, and endotoxin) to ensure safety. The CAR T cells were also labeled first with goat anti-mouse F(Ab’)2 antibody, then with streptavidin-PE and anti-human CD3-PerCp, and analyzed by flow cytometry, to determine transduction efficiency. NK cells were determined by flow cytometry using sCD3-CD56+/CD16+ gates while Treg cells were determined using CD3+CD4+CD127+C25+ gates.

Prior to transplantation, lymphodepletion (fludarabine 25 mg/m2/d and cyclophosphamide at 250 mg/m2/d) was conducted once daily on days -5, -4, and -3 before infusion. A dose escalation trial was conducted, where CAR T cells were provided in escalating doses of 2x10^6^-5x10^6^ cells per kilogram of bodyweight. The first three patients in the dose escalation trial are reported here.

### End points

Primary end points were number of adverse events after CD4-IL15/IL15sushi CAR T cell infusion (time frame: 2 years, with focus on first 28 days after infusion) to determine the toxicity profile of CD4-IL15/IL15sushi CAR T cells. Adverse events after CAR T cell infusion were graded according to National Institutes of Health criteria (Common terminology Criteria for Adverse Events, version 4). Secondary end points include incidence of treatment-emergent adverse events (time frame: up to 6 months), disease-free survival (time frame: up to 2 years), progression-free survival (time frame: up to 2 years), and overall survival (time frame: up to 2 years). Clinical response was determined using the mSWAT and Global Response Score for Patients 1 and 2. Clinical response was determined using RECIL criteria for Patient 3.

## Results

### Generation of the third generation CD4-IL15/IL15sushi CAR

**Figure 1:**
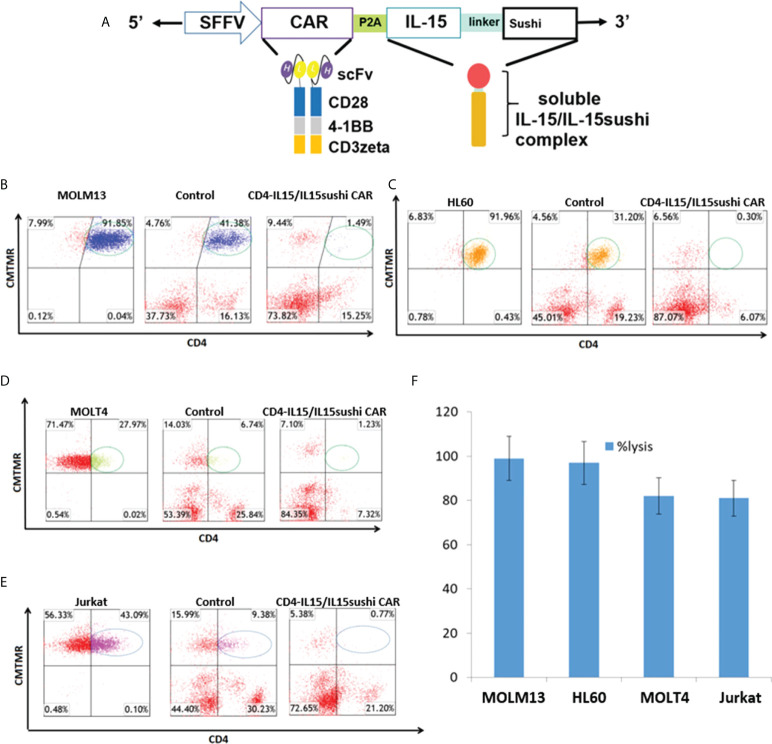
CD4-IL15/IL15sushi construct and *in vitro* validation. **(A)** Schematic representation of recombinant lentiviral vector encoding a third generation CD4 CAR linked with the P2A self-cleaving sequence to the IL-15/IL-15sushi domain of the IL-15 alpha receptor. Expression is driven by the spleen focus-forming virus (SFFV) promoter. The IL-15/IL-15sushi portion is composed of an IL-2 signal peptide fused to IL-15 and linked to the sushi domain *via* a 26-amino acid poly-proline linker. **(B)** Target MOLM13 cells expressing CD4 were co-cultured with control T cells (middle panel) or CD4-IL15/IL15sushi CAR T cells (right panel) at an E:T ratio of 2:1 for 24 hours. Left panel shows target cells alone. Target cells were pre-stained with CellTracker (CMTMR) to help distinguish from CD4-IL15/IL15sushsi CAR T cells. T cells are displayed in red. 97% of circled target population lysed compared to control in average of two experiments. **(C)** Target HL60 cells used in same experimental design. 99% of circled target population lysed compared to control in average of two experiments. **(D)** Target MOLT4 cells used in same experimental design. 82% of circled target population lysed compared to control in average of two experiments. **(E)** Target Jurkat cells used in same experimental design. 82% of circled target population lysed compared to control in average of two experiments. **(F)** Percent lysis of target population by CD4-IL15/IL15sushi CAR T cells compared to control T cells from **(B–E)**. Each bar represents the average of duplicate samples.

We have previously shown that CD4 CAR demonstrates cytotoxic effects against T cell malignancies (23, 24). Briefly, CD4 CAR is composed of a single-chain variable fragment (scFv) of an anti-CD4 molecule derived from the humanized monoclonal ibalizumab, which has been used in clinical trials for blocking HIV binding to CD4 (41–43). This CAR contains the leader sequence of CD8 for efficient surface expression, and the anti-CD4 scFv is linked to the intracellular domains by a CD8-derived hinge and transmembrane regions. CD4 CAR is a third-generation CAR, with two co-activation intracellular domains (CD28 and 4-1BB) fused to the CD3zeta T-cell activation signaling domain. To create CD4-IL15/IL15sushi CAR, this CD4 CAR was then linked to the IL-15/IL-15sushi domain by a P2A self-cleaving sequence. The IL-15/IL-15sushi domain consists of an IL-2 signal peptide fused to IL-15, which is linked to the soluble, sushi domain of the IL-15α receptor *via* a 26-amino acid poly-proline linker (**Figure 1A**). The construct was transduced into both T cells and NK92 cells (**Supplementary Figure 1**).

### CD4-IL15/IL15sushi CAR T cells lyse CD4+ cell lines *in vitro*

To assay the ability of the CD4-IL15/IL15sushi CAR T cells to target CD4+ cells, 24 h co-cultures with either control or CD4-IL15/IL15sushi CAR T cells versus four different CD4-expressing tumor cell lines were performed in an E:T ratio of 2:1. MOLM13 and HL60 (acute myeloid cell lines) target lines, which both had high surface expression of CD4+, were almost completely ablated by CD4-IL15/IL15sushi CAR T cells compared to control T cells (97% and 99% lysis, respectively) (**Figures 1B, C**). MOLT4 and Jurkat, acute lymphoblastic T cell lines with lower CD4 expression, were also co-cultured with CD4-IL15/IL15sushi CAR T cells or control T cells. The CD4-IL15/IL15sushi CAR T cells were able to lyse approximately 80% of the target, CD4+ population in each of these cell lines (**Figures 1D, E**). The results of these four experiments are summarized in **Figure 1F**. Similar results were obtained when CD4-IL15/IL15sushi CAR NK92 cells were used instead of T cells (**Supplementary Figure 2**).

### CD4-IL15/IL15sushi CAR T cells exhibit significant anti-tumor activity *in vivo*

**Figure 2:**
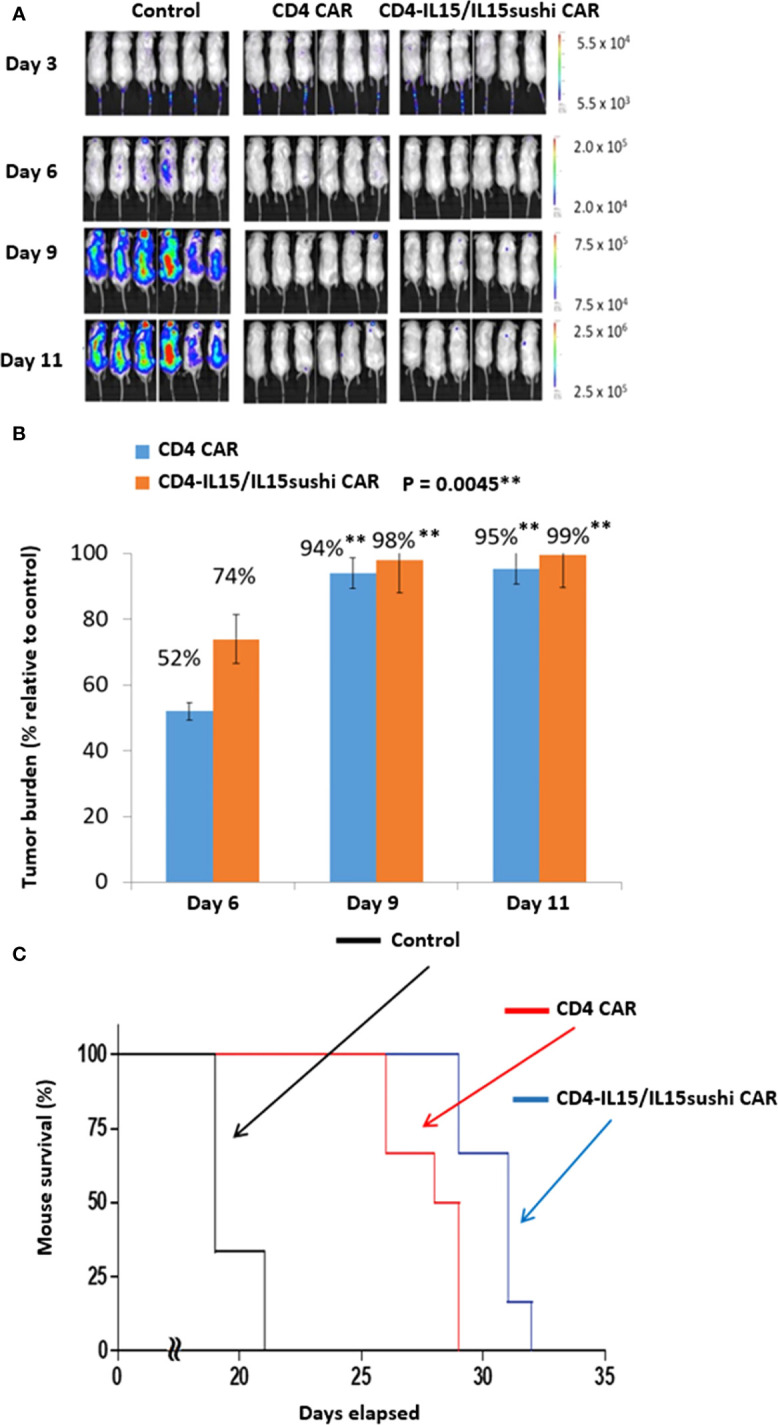
CD4 CAR and CD4-IL15/IL15sushi CAR T cells reduce tumor burden in MOLM13 mouse model. **(A)** NSG mice were sub-lethally irradiated and intravenously injected with 1.0x10^6^ luciferase-expressing MOLM13 cells, an acute myeloid leukemia cell line that is 100% CD4+ to induce measurable tumor formation. Three days following tumor cell injection, 6 mice were intravenously injected with a course of 8x10^6^ vector control, CD4 CAR, or CD4-IL15/IL15sushi CAR T cells. On days 3, 6, 9, and 11, mice were injected subcutaneously with RediJect D-luciferin and subjected to IVIS imaging to measure tumor burden. **(B)** Average light intensity (photons/sec) measured for CD4 CAR and CD4-IL15/IL15sushi was compared to that of control to determine the percentage of tumor burden in treated versus control mice. By Day 6, CD4 CAR-treated mice had 52% lower tumor burden relative to control while CD4-IL15/IL15sushi-treated mice had 74% lower. Unpaired t-test analysis revealed a significant difference between control and two treatment groups by Day 9 (P = 0.0045). By Day 11, tumor burden was nearly 100% decreased in both CD4 CAR and CD4-IL15/IL15sushi CAR groups compared to control. **(C)** Mouse survival was compared across the groups. Log-rank analysis revealed a significant difference in survival of the two treatment groups compared to the control (P = 0.0003). Additionally, the CD4-IL15/IL15sushi CAR treatment group had a significantly improved survival compared to the CD4 CAR treatment group (P = 0.0087). ** means a p-value =< 0.01, also called "very significant".

To evaluate the *in vivo* anti-tumor activity of the CD4-IL15/IL15sushi CAR T cells, we developed a xenogeneic mouse model using NSG mice sub-lethally irradiated (2.0 Gy) and intravenously injected with 1.0x10^6^ luciferase-expressing MOLM13 cells 24 hours later to induce measurable tumor formation. Three days following tumor cell injection, 6 mice each were intravenously injected with 8x10^6^ vector control, CD4 CAR, or CD4-IL15/IL15sushi CAR T cells. On days 3, 6, 9, and 11, mice were injected subcutaneously with RediJect D-luciferin (Perkin Elmer) and subjected to IVIS imaging to measure tumor burden (**Figure 2A**). Average light intensity determined that CD4 CAR T cell-treated mice had a 52% lower tumor burden relative to control on Day 6, whereas CD4-IL15/IL15sushi CAR T cells had a 74% lower tumor burden (**Figure 2B**). On Day 11, there was nearly 100% decreased tumor burden in both treatment groups compared to control. Unpaired t-test analysis revealed a very significant difference (P = 0.0045) between the control and two treatment groups by Day 9. Analysis of mouse survival revealed a very significant difference (P = 0.0003) between the control and two treatment groups as well (**Figure 2C**). Additionally, CD4-IL15/IL15sushi CAR T cell-treated group had a significant improvement in survival compared to CD4 CAR T cell-treated group (P = 0.0087), demonstrating added benefit of IL15/IL15sushi complex inclusion in the CAR construct (**Figure 2C**).

### CD4-IL15/IL15sushi CAR NK92 cells demonstrate improved outcomes in “stressed” *in vivo* environment

To further compare the function of the CD4-IL15/IL15sushi CAR and CD4 CAR, we created a “stressful” condition by using CD4-IL15/IL15sushi CAR NK92 cells and Jurkat tumors. NK92 cells have shorter persistence compared to T cells, and Jurkat cells have lower CD4 expression compared to MOLM13 cells (**Supplementary Figure 3A**). Both of these factors favor earlier tumor relapse.

**Figure 3:**
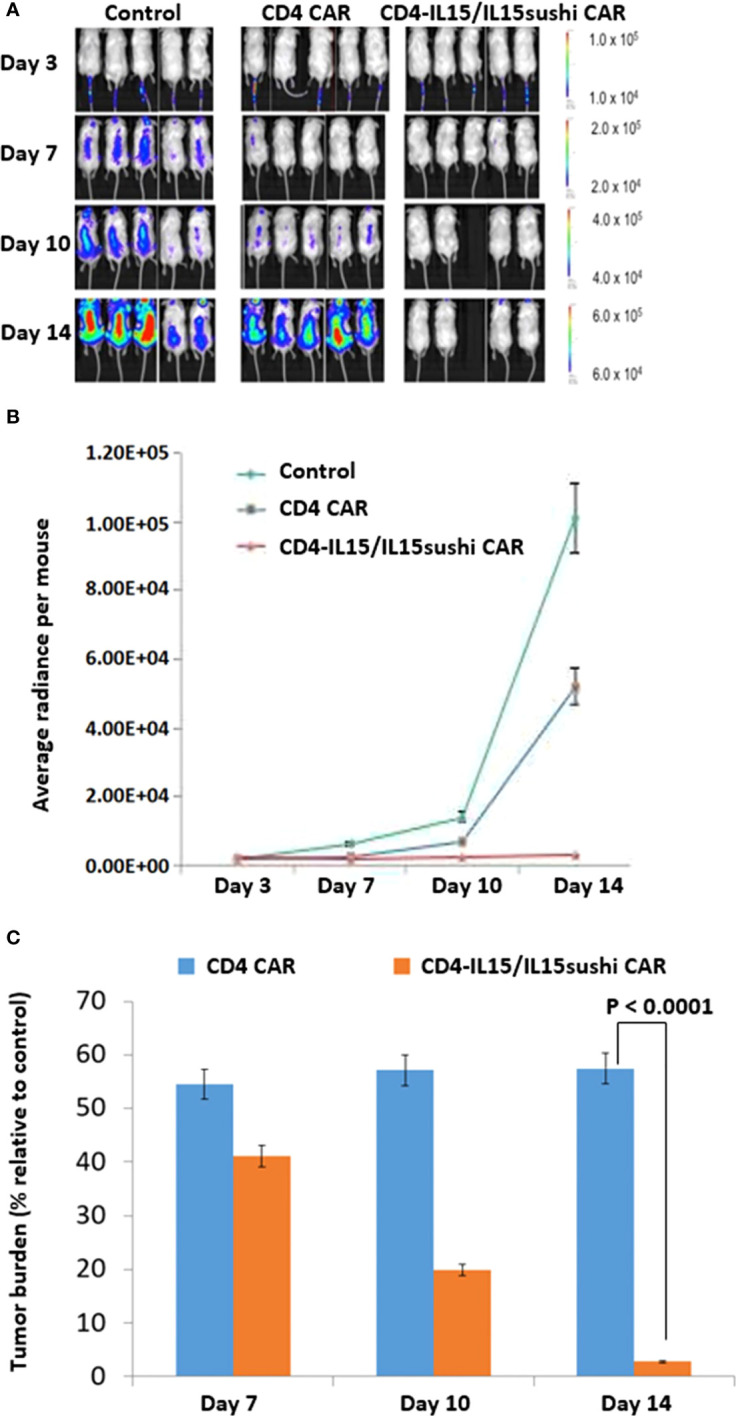
CD4-IL15/IL15sushi CAR NK92 cells reduce tumor burden in Jurkat mouse model. **(A)** NSG mice were sub-lethally irradiated and intravenously injected with 1.0x10^6^ luciferase-expressing Jurkat cells to induce measurable tumor formation. Three days following tumor cell injection, 5 mice were intravenously injected with a course of 10x10^6^ vector control NK92, CD4 CAR NK92, or CD4-IL15/IL15sushi CAR NK92 cells. On days 3, 7, 10, and 14, mice were injected subcutaneously with RediJect D-luciferin and subjected to IVIS imaging to measure tumor burden. One mouse died on Day 10 after NK92 injection, most likely due to injection procedure and NK92 cell aggregation. This mouse was sick immediately after injection. **(B)** Average light intensity for control NK92, CD4 CAR NK92, and CD4-IL15/IL15sushi CAR NK92 cells was measured in average total flux (photons/sec). CD4-IL15/IL15sushi CAR NK92 cells had lower light intensity than control and CD4 CAR groups. **(C)** Average light intensity measured for CD4 CAR and CD4-IL15/IL15sushi CAR was compared to that of control to determine the tumor burden in treated versus control mice. Although both conditions showed significant reduction in tumor burden by Day 7, relative tumor burden for CD4 CAR NK92 cells stayed the same to Day 14, while CD4-IL15/IL15sushi CAR NK92 cells continued to decrease by over 97%. Unpaired t-test analysis revealed an extremely significant difference (P < 0.0001) between the CD4 CAR NK92 and CD4-IL15/IL15sushi CAR NK92 treatment groups by Day 14.

We used a xenogeneic mouse model using NSG mice sub-lethally irradiated (2.0 Gy) and intravenously injected 24 hours later with 1.0x10^6^ luciferase-expressing Jurkat cells to induce measurable tumor formation. Three days after tumor injection, mice were intravenously injected with a course of 10x10^6^ vector control NK92 cells, CD4 CAR NK92 cells, or CD4-IL15/IL15sushi CAR NK92 cells. Mice were subjected to IVIS imaging to measure tumor burden on days 3, 7, 10, and 14 (**Figure 3A**). Measurement of average light intensity showed that both CD4 CAR NK92-treated and CD4-IL15/IL15sushi CAR NK92 significantly reduced tumor burden compared to control by Day 7 (**Figures 3B, C**). However, afterwards tumor burden remained relatively constant in the CD4 CAR group to Day 14, while tumor burden in the CD4-IL15/IL15sushi CAR group continued to decrease by over 97%. Unpaired t-test analysis of the radiance indicated an extremely significant difference (P < 0.0001) between the CD4 CAR and CD4-IL15/IL15sushi CAR groups by Day 14. This suggests that CD4-IL15/IL15sushi CAR may provide even greater benefit compared to CD4 CAR in conditions that favor early relapse and treatment failure.

## Clinical trial

CD4-IL15/IL15sushi CAR T cells were then tested in a pilot clinical phase I trial (NCT04162340). The results of the first three patients enrolled in the dose escalation trial are reported here. Patient 1 is a 54-year-old patient with relapsed refractory stage IVb Sézary syndrome, an aggressive CTCL. The patient had been having symptoms of erythroderma, pruritus, and scaling of the skin for over 10 years. Previous treatments with IFN-α, histone deacetylase inhibitor (HDACi), and multi-agent chemotherapy (gemcitabine, doxorubicin, and CHOP-like regimen) had all failed. The patient suffered from frequent respiratory and skin infections with extensive skin lesions. The patient was given a pretreatment regimen of fludarabine and cyclophosphamide (described in Methods) before CD4-IL15/IL15sushi CAR T cell infusion. The patient was then administered one dose of 2.8x10^6^ autologous CD4-IL15/IL15sushi CAR T cells/kg.

**Figure 4:**
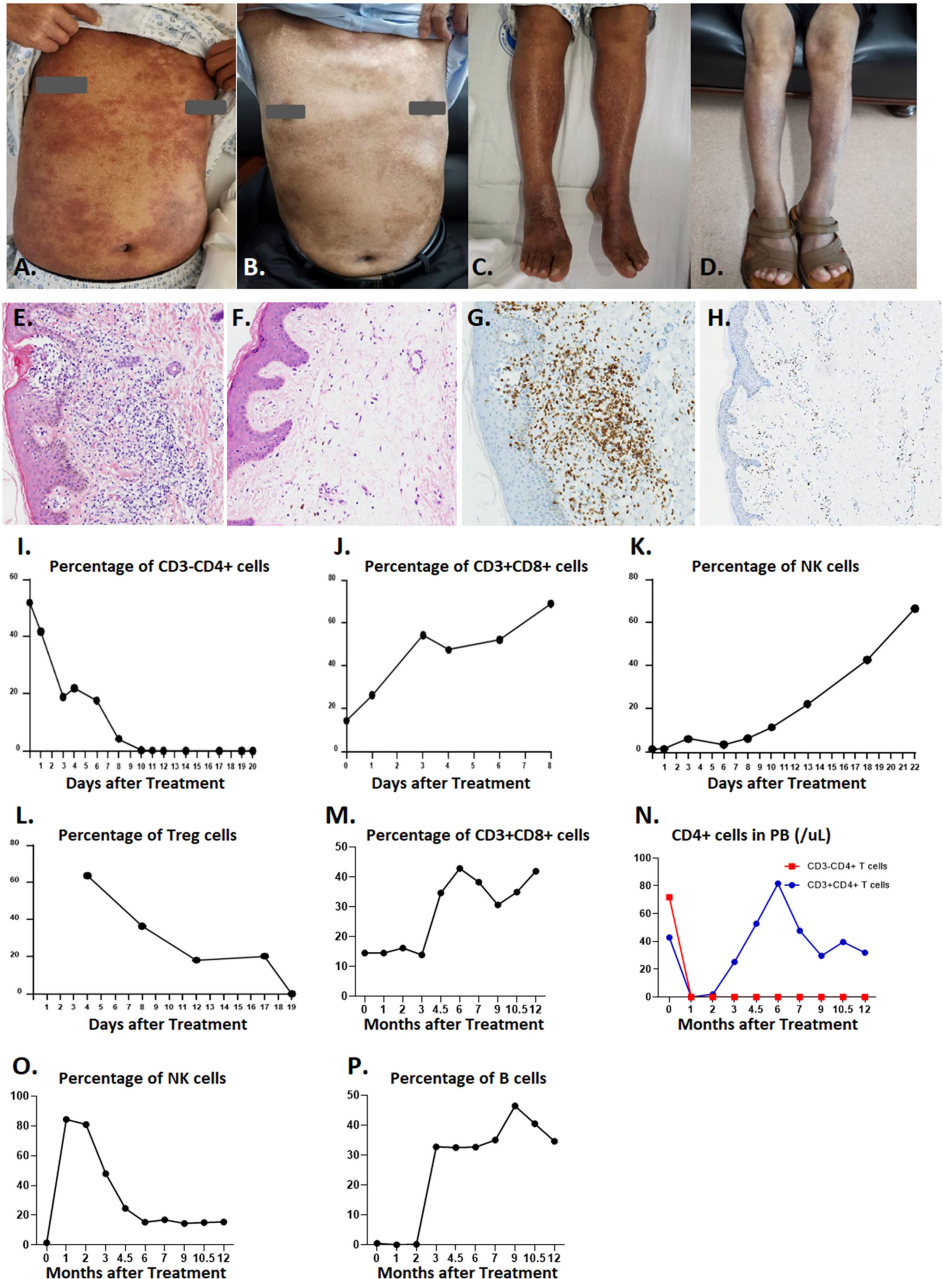
Efficiency of CD4-IL15/IL15sushi CAR T cells in Patient 1 with Sézary syndrome. **(A)** Chest skin appearance with marked erythema and swelling before treatment with CD4-IL15/IL15sushi CAR T cells. **(B)** Chest skin appearance on Day 28 post-infusion. **(C)** Leg skin appearance before treatment with CD4-IL15/IL15sushi CAR T cells. **(D)** Leg skin appearance on Day 28 post- infusion. **(E)** H&E staining of skin biopsy before treatment with CD4-IL15/IL15sushi CAR T cells, showing extensive lymphocytic infiltration. **(F)** H&E staining of skin biopsy 28 days after treatment, showing significantly diminished lymphocyte levels. **(G)** CD4 expression of leukemic Sézary cells in skin biopsy before treatment. **(H)** Diminished CD4 expression in skin biopsy 28 days after treatment. **(I)** CD3-CD4+ leukemic Sézary cells dramatically diminished within 20 days after infusion with CD4-IL15/IL15sushi CAR T cells. **(J)** Expansion of CD3+CD8+ cells within 8 days after infusion with CD4-IL15/IL15sushi CAR T cells. **(K)** Expansion of NK cells within 22 days after infusion. **(L)** Decreased Treg cells within 19 days after infusion. **(M)** Long-term expansion of CD3+CD8+ cells 1 year after infusion. **(N)** Decreased CD3-CD4+ cell counts in peripheral blood remained undetectable for the next year after treatment (orange curve), whereas CD3+CD4+ cells were depressed during the first month post-treatment followed by recovery in the next few months (blue curve) after treatment, which indicates that immune reconstitution can be observed post-treatment. **(O)** Transient expansion of NK cells to 84.41% during the first month post-treatment following by a gradual decrease until 6 months and maintenance of that level for 6 more months. **(P)** Initial suppression of B cells for 2 months followed by expansion.

While the patient had extensive skin lesions, covering >80% of total skin area prior to CD4-IL15/IL15sushi CAR T cell treatment (**Figures 4A, C**), the patient’s skin showed remarkable improvement 28 days post-therapy (**Figures 4B, D**). Skin biopsy before treatment showed extensive lymphocytic infiltration of CD4+ cells (**Figures 4E, G**), which was cleared in a repeat skin biopsy 28 days after infusion (**Figures 4F, H**). Molecular testing for T-cell gene rearrangement (**Supplementary Figures 3A, B**), flow cytometry of peripheral blood (**Supplementary Figures 3C, D**), and virtual absence of CD3+ on skin biopsy (**Supplementary Figures 3E, F)** further confirm the complete molecular remission of this patient’s Sézary syndrome.

CD4-IL15/IL15sushi CAR T cells demonstrated potent targeted lysis of Sézary leukemic T cells, which were CD3-CD4+ in this patient. While the percentage of Sézary leukemic cells detected in peripheral blood prior to CD4-IL15/IL15sushi CAR T cell therapy was about 50%, the leukemic cells were undetectable by Day 10 after treatment (**Figure 4I**). CD3+CD8+ cells markedly expanded from about 18% to 70% of lymphocytes in the first week post-infusion (**Figure 4J**). NK expansion followed CD8+ T cell expansion and reached about 65% of lymphocytes on Day 22 post-infusion (**Figure 4K**). Concurrently, the immunosuppressive CD4+ Treg cells were suppressed in the first month following infusion (**Figure 4L**).

Long-term observation of the peripheral blood populations showed elevated percentage of CD3+CD8+ cells 1 year after treatment (**Figure 4M**). Within a month after treatment, both normal CD3+CD4+ and leukemic CD3-CD4+ declined to undetectable levels (**Figure 4N**). While the CD3-CD4+ T cells remained low 1 year after treatment, the CD3+CD4+ T cell levels began to rise 3 months after treatment (**Figure 4N**), indicating the remission of leukemic cells and the regeneration of normal CD4+ cells, presumably due to loss of CD4-IL15/IL15sushi CAR T cell functional persistence. While the NK cell levels had risen immediately after therapy, they began to decline to a normal level after 2 months (**Figure 4O**). Additionally, B cells, which had been suppressed during the first few months of treatment, expanded after 3 months (**Figure 4P**). Taken together, these results demonstrate the ability of CD4-IL15/IL15sushi CAR T cells to transiently alter the immune milieu, resulting in the complete remission of Sézary syndrome in this patient. Patient 1 was found to be in complete remission (CR) by mSWAT and Global Response Scoring and has remained in CR for 15 months.

Patient 2 is a 45-year-old female with the CTCL mycosis fungoides lymphoma (stage IVb) with 9 years of skin erythema. Previous lines of treatment, including steroids, immunomodulatory drugs (IMiDs), immunosuppressors, retinoic acid, CHOP-based chemotherapy, and HDACi, had all failed. After a liposome-entrapped mitoxantrone (LEM) clinical trial failed, the disease was considered refractory, and the patient was enrolled in this trial. The patient received fludarabine and cyclophosphamide pretreatment regimen followed by a split dose of CD4-IL15/IL15sushi CAR T cells of 2.0x10^6^ cells/kg and 1.2x10^6^ cells/kg (3.2x10^6^ cells/kg total).

**Figure 5:**
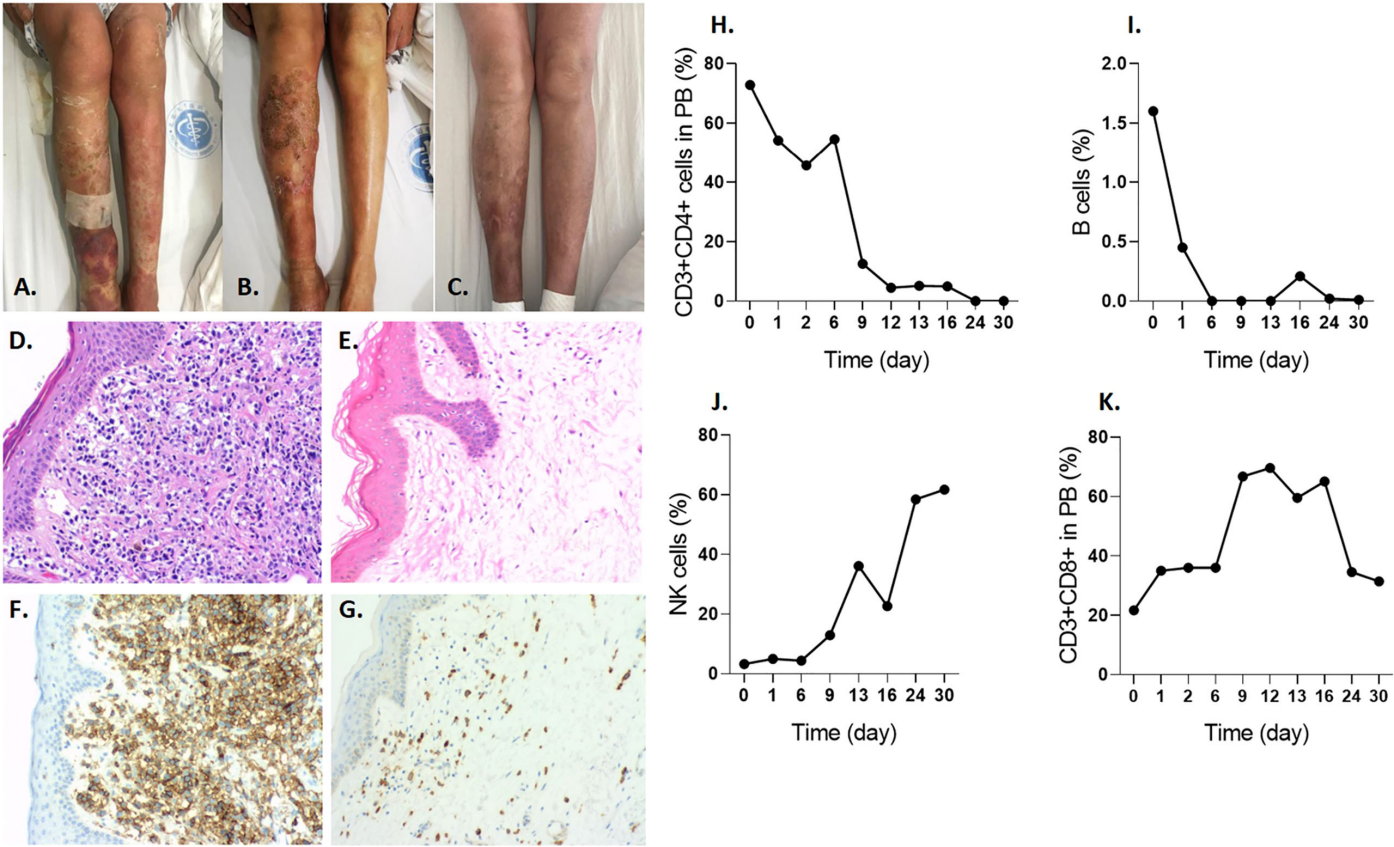
CD4-IL15/IL15sushi CAR T cells improve symptoms in Patient 2 with mycosis fungoides lymphoma. **(A)** Leg skin appearance with erythema and swelling before treatment with CD4-IL15/IL15sushi CAR T cells. **(B)** Leg skin appearance 2 weeks after treatment shows some improvement. **(C)** Leg skin appearance after 4 weeks after treatment show even further improvement. **(D)** H&E staining of skin biopsy before treatment show numerous lymphocytic infiltrates. **(E)** H&E staining of skin biopsy days after infusion of CD4-IL15/IL15sushi CAR T cells show less lymphocytic cells in the skin. **(F)** CD4 expression of malignant T cells in skin biopsy before treatment. **(G)** Diminished CD4 expression in skin biopsy days after infusion. **(H)** CD3+CD4+, including the malignant cells, reduced to undetectable levels by 24 days after CD4-IL15/IL15sushi CAR T cell therapy. **(I)** Therapy led to rapid suppression of B cells to undetectable levels by 6 days after treatment. **(J)** Expansion of NK cells 1 month after therapy. **(K)** Expansion of CD3+CD8+ cells in the first month post-infusion.

Imaging of the patient’s skin before infusion (**Figure 5A**), 2 weeks after infusion (**Figure 5B**), and 4 weeks after infusion (**Figure 5C**) revealed a significant improvement due to CD4-IL15/IL15sushi CAR T cell therapy. Skin biopsy before therapy revealed intensive CD4+ lymphoma cell infiltrates (**Figures 5D, F**), which showed significant improvement 28 days post-infusion (**Figures 5E, G**). Unlike the Patient 1, this patient’s malignant cells were CD3+CD4+. CD3+CD4+ cells, representing both malignant and normal CD4+ cells in this patient, declined to undetectable levels by Day 24 post-therapy (**Figure 5H**). B cells also decreased to undetectable levels by Day 24 post-infusion (**Figure 5I**). On the other hand, this patient had a profound expansion of NK cells (**Figure 5J**) and CD3+CD8+ cells (**Figure 5K**) during the first month. The patient was found to be in partial remission (PR) as assessed by mSWAT and Global Response Scoring and remains in PR 5 months post-treatment.

**Figure 6:**
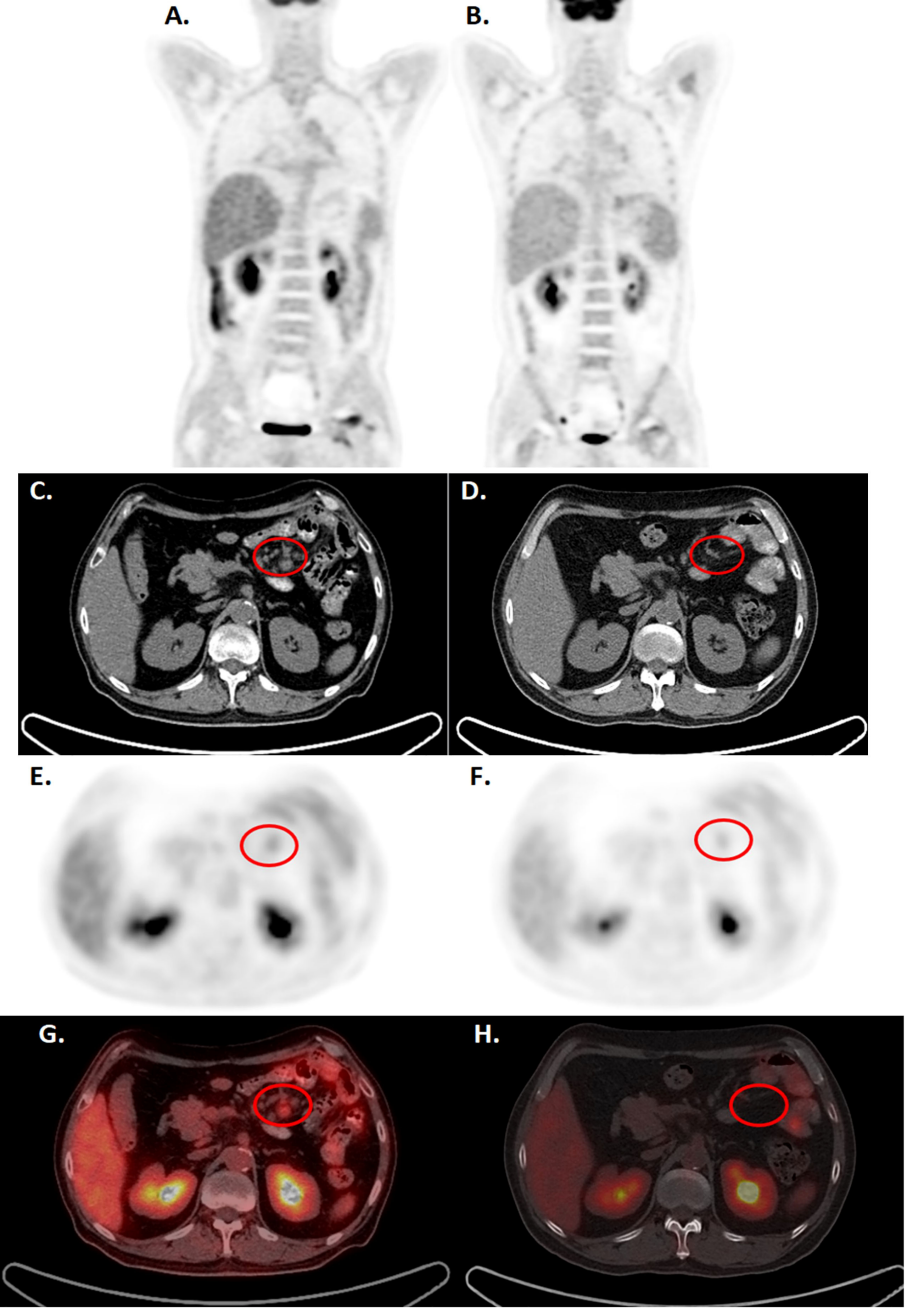
CD4-IL15/IL15sushi CAR T cells led to clinical improvements in Patient 3, diagnosed with mesenteric lymphoma, by PET-CT. **(A, C, E, G)** PET-CT before CD4-IL15/IL15sushi CAR T cell therapy show significant FDG uptake in mesenteric lymph nodes (circled). The larger retroperitoneal and mesenteric lymphadenopathy measure 2.4*1.7 cm, with the SUVmax of 2.1-5.2 **(B, D, F, H)** PET-CT 11 weeks after therapy showed reduction of mesenteric lymph node size and FDG avidity. The proto-enlarged lymph node lesions were significantly reduced or absent from before, with SUVmax of 0.9.

Patient 3 is a 57-year-old male diagnosed with the PTCL angioimmunoblastic T cell lymphoma in mesenteric lymph nodes. The patient failed multiple lines of treatments, including steroids, CHOP-based chemotherapy, gemcitabine, proteasome inhibitors, and HDACi. The patient had suffered from severe virus, fungi, and bacterial infections, and the tumor progressed with multiple lymph node enlargement and intestinal infiltrations, associated with fever, diarrhea, and wasting. The patient received pretreatment fludarabine and cyclophosphamide regimen and received 3.5x10^6^ cells/kg of CD4-IL15/IL15sushi CAR T cells in two split doses (2x10^6^ cells/kg and 1.5x10^6^ cells/kg). PET-CT demonstrated marked reduction in mesenteric lymph node size and FDG avidity at 11 weeks post-infusion (**Figure 6**). Similar reductions were observed on CT 2 weeks and 24 weeks post-infusion (**Supplementary Figure 4**). These scans demonstrate the ability of CD4-IL15/IL15sushi CAR T cells to ablate the malignant cells within the mesenteric lymph nodes. Peripheral blood studies demonstrated similar trends observed for Patients 1 and 2, reflecting decreases in CD3+CD4+ associated with expansion in the CD3+CD8+ and NK cell populations (**Supplementary Figure 5**), demonstrating the killing efficiency of CD4-IL15/IL15sushi CAR. Patient 3 was found to be in CR as assessed by the RECIL criteria and remains in CR for 8 months.

Two patients (Patients 1 and 3) experienced a Grade II CRS toxicity and one patient (Patient 2) experienced Grade I CRS. No patients experienced neurotoxicity during treatment. No patients experienced any severe opportunistic infections. Patients 1 and 2 did not receive any prophylactic antibiotics or antifungal therapy during treatment. Patient 3 did have recurrent Epstein-Barr viremia prior to treatment and following treatment, and it was uncertain whether this was treatment-related. As a precaution, Patient 3 was started on oral trimethoprim-sulfamethoxazole and oral ganciclovir with no further occurrences. Patient 3 also had RBC reduction that required blood transfusion, which recovered within 8 weeks. Full toxicity details are reported in **Table 1**. Overall, CD4-IL15/IL15sushi CAR T cells were well-tolerated in all patients.

**Table 1:** Toxicity profile of CD4-IL15/IL15sushi CAR T cells.

Classification of adverse toxicity reactions		All levels (number of patients)	Level 1 or 2 (number of patients)	Level 3 or 4 (number of patients)
Hematologic toxicity	Anemia	2	1	1
	Leukopenia	2	1	1
	Neutropenia	2	1	1
	Lymphocytopenia	3	2	1
	Thrombocytopenia	1	1	0
Infection	Transient Epstein-Barr viremia	1	1	0
	Other opportunistic infections	0	0	0
Electrolyte	Hypercalcemia	0	0	0
	Hypocalcemia	0	0	0
	Hypokalemia	0	0	0
	Hyponatremia	0	0	0
Central Nervous System		0	0	0
Urinary System		0	0	0
Respiratory System		1	1	0
Digestive System		1	1	0
Other Systems		0	0	0
Brief Summary		13	9	4

Toxicity profile demonstrates that toxicity experienced is predominantly level 1 or 2 and predominantly limited to the hematological system.

**Figure 7:**
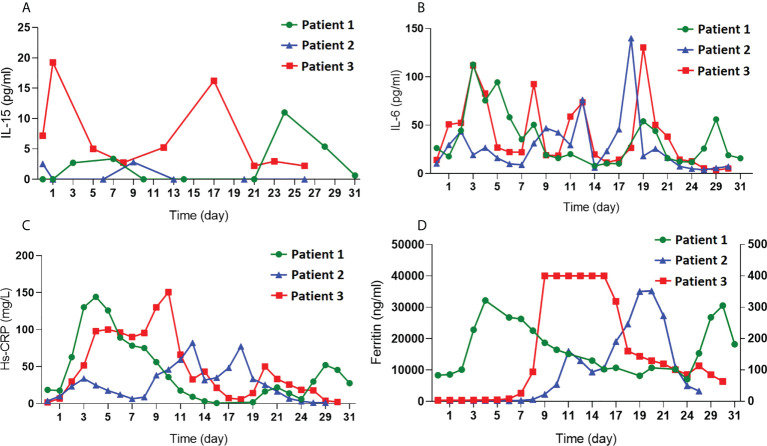
Measurement of cytokines and acute phase reactants after CD4-IL15/IL15sushi CAR T cell infusion. **(A)** Measurement of IL-15, **(B)** IL-6, **(C)** Hs-CRP, and **(D)** ferritin in the blood of all three patients demonstrate low levels of IL-15 despite secretion of IL15/IL15sushi complex from CD4-IL15/IL15sushi CAR T cells and demonstrate transient, mild increases in acute phase reactants.

As CD4-IL15/IL15sushi CAR T cells secrete the IL15/IL15sushi complex, serum IL-15 was measured in the patients’ blood samples during the first month post-treatment to determine whether CD4-IL15/IL15sushi CAR leads to high IL-15 loads. Serum IL-15 levels was very low (<20 pg/mL) and within normal limits in all three patients in the first month post-infusion (**Figure 7A**). Markers for acute phase reactants, including IL-6, Hs-CRP, and ferritin, which were also measured in the first month post-infusion, were transiently elevated after CAR administration (**Figures 7B–D**). These results show that CD4-IL15/IL15sushi CAR T cells do not significantly alter serum levels of various cytokines/proteins.

## Discussion

Despite recent advances in hematologic cancer treatments, prognosis for advanced PTCLs and CTCLs remains poor with limited treatment options. The positive results of CD19 CAR T cells against B cell malignancies has encouraged the development of CAR T cells against other cancers, such as T-cell and non-hematological malignancies. CD7 CAR led to the complete remission of 90% (n = 18) of relapsed or refractory T-ALL, with remission maintained at a median follow-up of 6.3 months in 15 patients (11). CD5 CAR T cells have also shown promising early clinical results, with 4/9 patients achieving an objective response to CD5 CAR T cells, and CD5-IL15/IL15sushi CAR T cells leading to the remission of CNS T-LBL in one patient (13, 14).

We demonstrate here that CD4-IL15/IL15sushi CAR cells, another potential target for T-cell malignancies, exhibited effective targeting and potent lysis of CD4+ cells *in vitro* and *in vivo*. Additionally, CD4-IL15/IL15sushi CAR cells outperformed CD4 CAR cells *in vivo*, reducing tumor burden and providing survival benefit, suggesting that the secreted IL15/IL15sushi complex may be beneficial in the treatment of T-cell malignancies. Given the superior results of CD4-IL15/IL15sushi CAR T cells in these *in vivo* experiments, this construct was then used in a phase I trial against refractory PTCL/CTCL, with the first three patients in the dose escalation trial demonstrating complete remission without the development of severe adverse effects.

CD4-IL15/IL15sushi CAR is expected to lead to a transient CD4+ T-cell aplasia, similar to the B-cell aplasia seen with CD19 CAR T cells. Clinical trials of monoclonal CD4 antibodies have reported low frequencies of severe infections despite profound decreases in CD4+ cell levels, and CD4 levels began to rise soon after completion of treatment (16–22). Similar T-cell aplasia has been observed in clinical trials of CD7 and CD5 CAR. CD7 CAR led to temporary neutropenia with a median time from infusion to recovery of absolute neutrophil count of 1,000/μL of 60 days (10). 20% (4/20) of the patients had viral reactivations that resolved with antiviral therapy, and 1 patient with a pre-enrollment history of fungal infection died of pulmonary hemorrhage associated with fungal pneumonia 5.5 months post-therapy (10). CD5 CAR led to prolonged cytopenia in 2/9 patients at 6 weeks in a dose escalation trial (13). In a previous study we performed on CD5-IL15/IL15sushi CAR T cells, CD5+ T cells returned to normal levels nine days post-infusion (14). Similarly, CD4-IL15/IL15sushi CAR T cells led to the transient ablation of CD4+ T cells in Patient 1, with recovery at 3 months.

Importantly, CD3+CD8+ T cells and NK cells expanded in the patients, which may have contributed to the lack of severe infection during the period of CD4+ aplasia. This CD3+CD8+ T cell and NK cell lymphocytosis may be due to the ability of CD4-IL15/IL15sushi CAR T cells to target Treg cells. Treg cells, which are CD4+, were noted to be remarkably suppressed in the first month post-transfusion in Patient 1. As Treg cells are normally potent inhibitors of immune activation and are often coopted by cancers to escape immune-mediated destruction, elimination of Treg cells may be an unintended benefit of CD4-IL15/IL15sushi CAR T cells. Without normal inhibition, CD3+CD8+ T cells and NK cells may have been free to expand to relative high levels despite pre-CAR therapy with immunosuppressive drugs. In addition, Treg cells have been shown to inhibit cytotoxicity mediated by both CD8+ T cells and NK cells (44). Inhibition of Treg may thus not only have increased relative proportions of these immune cells but allowed them to be more functionally active, offering greater protection for the patients. Alternatively, the secretion of IL-15 by CD4-IL15/IL15sushi CAR T cells may have been beneficial to the host immune response as IL-15 has been shown to increase NK and T cell expansion and persistence, which may help explain the increased relative proportions of CD8+ T cells and NK cells observed in Patient 1 (28, 29). These changes in CD8+ T cell and NK cell levels and functioning have been reported in clinical studies of recombinant IL-15 injections, which resulted in favorable tumor responses (37, 39, 40). Further studies are needed to more fully explore whether this CD3+CD8+ and NK lymphocytosis could be due to Treg suppression and/or secretion of IL-15. In addition to the possible contributions of Treg suppression and/or IL-15 secretion on host immune functioning, CD3+CD8+ T cells may have been spared relatively more than would be expected compared to other T-cell directed therapies. While pan-T-cell marker directed therapies would also attack CD8+ T cells, since CD8+ T cells are CD4- they would not be targeted by CD4-IL15/IL15sushi CAR T cells, allowing for the CD8+ population to escape CAR-mediated destruction. While CD3+CD8+ T cell and NK cell expansion and/or maintenance may lead to decreased risk of infection compared with other T-cell directed CAR strategies, future studies are needed to further compare the risk with each construct.

Despite the recovery of CD4+ T cells at around 3 months, CD3- CD4+ Sézary cells remained undetectable one year after treatment. This prolonged suppression of CD3-CD4+ and recovery of CD3+CD4+ T cells could indicate that CD4-IL15/IL15sushi CAR T cells initially depleted both populations of CD4+ cells. After a few months, the population of CD4-IL15/IL15sushi CAR T cells may have become functionally inactive, allowing for the regeneration of normal CD4+ from CD4- hematopoietic precursors, which would have been spared by CD4-IL15/IL15sushi CAR T cell therapy. In contrast, malignant Sézary cells originate from clonal, mature CD4+ T cells, which would not have been spared by CD4-IL15/IL15sushi CAR T cells. Therefore, CD4-IL15/IL15sushi CAR T cells may have effectively reset the CD4 population, allowing regeneration of normal cells from hematopoietic stem cells while eliminating cells that could give rise to additional Sézary cells, preventing as a robust recovery.

CAR therapy is often associated with CRS toxicity as an adverse effect due to widespread immune activation as CAR cells encounter the malignant cells and are subsequently stimulated, resulting in elevated levels of inflammatory cytokines. The risk of CRS might be more substantial in CAR cells which secrete cytokines, such as CD5-IL15/IL15sushi CAR T cells. Additionally, high levels of IL-15 have been associated with uncontrolled lymphocytic proliferation (45). However, the CD4-IL15/IL15sushi CAR T cells were well-tolerated in the three patients, with two patients experiencing Grade II CRS and one patient Grade I CRS. Additionally, the total serum IL-15 was within normal limits in all three patients. Similarly, a phase I and II trial of NK cells transduced with both CD19 CAR and IL-15 demonstrated no increase in IL-15 levels over baseline (46). In contrast, direct injections of IL-15 or IL-15 complexes have led to greatly increased serum levels of IL-15 in clinical trials (37–40). These suggest that CAR cells may be used as vehicles to deliver the cytokines locally to the tumor microenvironment where it can provide the most benefit while avoiding the potential toxicities associated with high systemic IL-15 levels.

Further studies are needed to determine the optimal dosing of CD4-IL15/IL15sushi CAR to balance the risk of infection/CRS with antitumor effect. While CD4-IL15/IL15sushi CAR T cells appear to be safe in this preliminary study, the potential for prolonged T-cell aplasia or severe CRS remains. To mitigate this concern, we are currently working on a construct that contains two rituximab-binding epitopes in the hinge region of CD4-IL15/IL15sushi CAR. A rituximab-binding safety switched has been incorporated in CARs previously, without alteration in CAR functionality and with effective depletion when provided rituximab *in vivo* (47).

Traditionally, CAR T cells are comprised of CD4+ and CD8+ populations. However, CD4 CAR T cells are composed of CD8+ T cells only, due to fratricide of CD4+ T cells. Preclinical trials have demonstrated that effector CD8+ T cells derived from memory T cells have the ability to persist, reform memory populations, and are capable of self-renewal (48, 49). We have previously shown that CD4 CAR T cells display this central memory phenotype after expansion (23), which may allow for CD4-IL15/IL15sushi CAR T cells to persist and maintain antitumor efficacy despite the absence of CD4+ T cells. Additionally, recent clinical studies have also shown that CD8+ CAR T cells alone can lead to a durable response in patients with multiple myeloma or B-cell NHL (50, 51). These preliminary studies demonstrate that the CD8+ CD4 CAR T cells may be sufficient to successfully eradicate tumors, although more studies on efficacy of sole CD4+ CAR T cells is needed.

Our clinical trials also show a marked reduction in Treg cells following CD4-IL15/IL15sushi CAR T cell infusion. As Treg cells are powerful immunosuppressants often utilized by tumor cells to prevent immune-mediated destruction, cancers associated with Treg maintenance and proliferation are often associated with worse outcomes (26). Targeting PD-L1 and PD-1, which mediate the Treg-tumor interaction leading to Treg proliferation, has been an active area of research, and there are currently six monoclonal antibodies targeting either PD-L1 or PD-1 that have shown anti-tumor efficacy in more than twenty cancer types (52). However, these inhibitors do not work for all cancer types, and some Phase III trials have failed to reach primary endpoints despite showing promising results in Phase I/II trials (53). CD4 CAR T cells may be a useful adjunct in the treatment of solid tumors by providing an additional mechanism of suppressing Tregs, enhancing the immune response against tumors.

In conclusion, the use of CD4-IL15/IL15sushi CAR T cells led to remission (2 CR, 1 PR) in three patients with PTCL/CTCL without serious adverse effects in a phase I clinical trial. CD4-IL15/IL15sushi CAR T cell infusion led to suppression of Treg cells and relative expansion of CD3+CD8+ T cells and NK cells. These factors may make the associated T-cell aplasia more tolerable in a CD4-based CAR therapy compared to therapies targeting pan T-cell markers. CD4-IL15/IL15sushi CAR T cells demonstrated favorable safety profiles, with tolerable CRS and no severe opportunistic infections. These results show that CD4-IL15/IL15sushi CAR T cells may be a promising future therapy for relapsed or refractory T-cell malignancies, and they may even have potential in supplementing solid tumor eradication through their ability to target Treg cells.

## Data availability statement

The original contributions presented in the study are included in the article/**Supplementary Material**. Further inquiries can be directed to the corresponding authors.

## Ethics statement

The studies involving human participants were reviewed and approved by Institutional Review Board of Peking University Shenzhen Hospital. The patients/participants provided their written informed consent to participate in this study. The animal study was reviewed and approved by Institutional Review Board of Stony Brook University. Written informed consent was obtained from the individual(s) for the publication of any potentially identifiable images or data included in this article.

## Author contributions

JF performed the research, analyzed data, and wrote the paper. HX, ZW, WZ, and LiS performed research and analyzed data. AC analyzed data and wrote the paper. QC and LT analyzed data. LeS performed CT and PETCT image processing and analyzed data. KP, WM, XJ, WH, and YM designed the CAR and conducted preclinical studies. HZ conceived of the study, designed the study, and collected the data. All authors contributed to the writing and revisions.

## Funding

Supported in part by grants from the National Science Foundation of China (No. 82100210 and No. 82170191), Shenzhen Science and Technology Planning Project (JCYJ20210324105802007), the National Science Foundation of Guangdong Province (No. 2021A1515012185), and Zhongshan Kefa (2019, No. 187), as well as research funds from iCAR Bio Therapeutics Ltd and iCell Gene Therapeutics LLC.

## Funding

Authors AC, KGP, MW, WMH, and YM (all with iCell) designed the CAR and conducted preclinical studies.

## Acknowledgments

The authors thank Todd Rueb and Rebecca Connor for valuable assistance with flow cytometry methods and live cell sorting.

## Conflict of interest

YM is a founder of iCell Gene Therapeutics LLC. AC, KP, WM, XJ, WH are employed by iCell Gene Therapeutics LLC.

The remaining authors declare that the research was conducted in the absence of any commercial or financial relationships that could be construed as a potential conflict of interest.

## Publisher’s note

All claims expressed in this article are solely those of the authors and do not necessarily represent those of their affiliated organizations, or those of the publisher, the editors and the reviewers. Any product that may be evaluated in this article, or claim that may be made by its manufacturer, is not guaranteed or endorsed by the publisher.

## Supplementary material

Supplementary Figure 1 CD4-IL15/IL15sushi Expression. **(A)** HEK-293FT cells were transfected with an empty vector to serve as a control (lane 1) or CD4-IL15/IL15sushi CAR (lane 2) lentiviral plasmids. Forty-eight hours after transfection, supernatant was removed, and cells were collected for Western blot analysis (left) with mouse anti-human CD3ζ antibody. Activated T cells from cord blood buffy coat were transduced with either an empty vector (control) or CD4-IL15/IL15sushi CAR viral supernatant from transfected HEK-293FT cells. Cells were stained with goat-anti-mouse F(Ab’) transduced with an empty vector control (middle) or CD4-IL15/IL15sushi CAR (right) and analyzed by flow cytometry. (B) NK92 cells were transduced similarly and analyzed by flow cytometry.

Supplementary Figure 2 CD4-IL15/IL15sushi CAR NK92 cells can ablate CD4+ tumors in vitro. T cells are in red. All target populations are circled and in green. **(A)** MOLT4 cells (right-most panel) were co-cultured with control NK92 cells, CD4 CAR NK92 cells, and CD4-IL15/IL15sushi CAR NK92 cells at an E:T ratio of 1:1 (top) and 2:1 (bottom) for 24 hours. CD4-IL15/IL15sushi CAR NK92 cells were able to lyse more target cells compared to CD4 CAR NK92 cells (84% vs. 85% at 1:1 ratio). **(B)** Similarly, Karpas cells (top left panel) were co-cultured with control NK92, CD4 CAR NK92, or CD4-IL15/IL15sushi CAR NK92 cells at E:T ratio of 5:1 for 24 hours. CD4-IL15/IL15sushi CAR NK92 cells again showed more potent lysis of Karpas cells compared to CD4 CAR NK92 cells (95% vs. 82%).

Supplementary Figure 3 Other findings in Patient 1 with Sézary syndrome. **(A)** T cell receptor (TCR) gene rearrangement before treatment with CD4-IL15/IL15sushi CAR T cells, demonstrating monoclonal rearrangement. **(B)** TCR rearrangement 5 months after infusion shows polyclonality with random pattern of distribution. **(C)** Flow cytometry results before treatment showing 51.88% CD3-CD4+ leukemia cells. **(D)** Flow cytometry 13 days post-therapy showing nearly undetectable levels of CD3-CD4+ leukemia cells. **(E)** CD3 expression in skin biopsy before treatment. CD3 expression is positive in these malignant cells as the immunohistochemistry staining detected intracellular CD3 found in the malignant cells (rather than just surface expression detected by flow cytometry). **(F)** CD3 expression in skin biopsy 28 days after treatment. **(G)** CD8 expression in skin biopsy before treatment. **(H)** CD8 expression in skin biopsy 28 days after treatment.

Supplementary Figure 4 CT scan of Patient 3 with angioimmunoblastic T cell lymphoma in mesenteric lymph nodes showing improvement after CD4-IL15/IL15sushi CAR T cell therapy. **(A, D, G)** CT scan before therapy. **(B, E, H)** CT scan on week 2. **(C, F, I)** CT scan on week 24 post-therapy. CT showed the gradual reduction and disappearance of multiple enlarged mesenteric lymph nodes after CD4-IL15/IL15sushi CAR T cell infusion.

Supplementary Figure 5 CD4-IL15/IL15sushi CAR T cells alter the peripheral blood makeup in Patient 3 with angioimmunoblastic T cell lymphoma in mesenteric lymph nodes. **(A)** CD3+CD4+ cells show a marked decline after infusion of CD4-IL15/IL15sushi CAR T cells (percentage of lymphocytes). **(B)** The absolute number of CD3+CD4+ cells measured declined in first month post-infusion. **(C)** CD3+CD8+ cells showed marked expansion following therapy (percentage of lymphocytes). **(D)**The absolute number of CD3+CD8+ cells measured expanded during the first few months after infusion. **(E)** NK cells demonstrated marked expansion in the first month following treatment before returning to baseline levels. **(F)** B cells demonstrated variable levels following CD4-IL15/IL15sushi CAR T cell treatment.
