# Supplementary figures and images for: Treatment of aggressive T-cell lymphoma/leukemia with anti-CD4 CAR T cells
Source: Front Immunol. 2022 Sep 12;13:997482. doi: 10.3389/fimmu.2022.997482 (PMC9511023; doi:10.3389/fimmu.2022.997482)

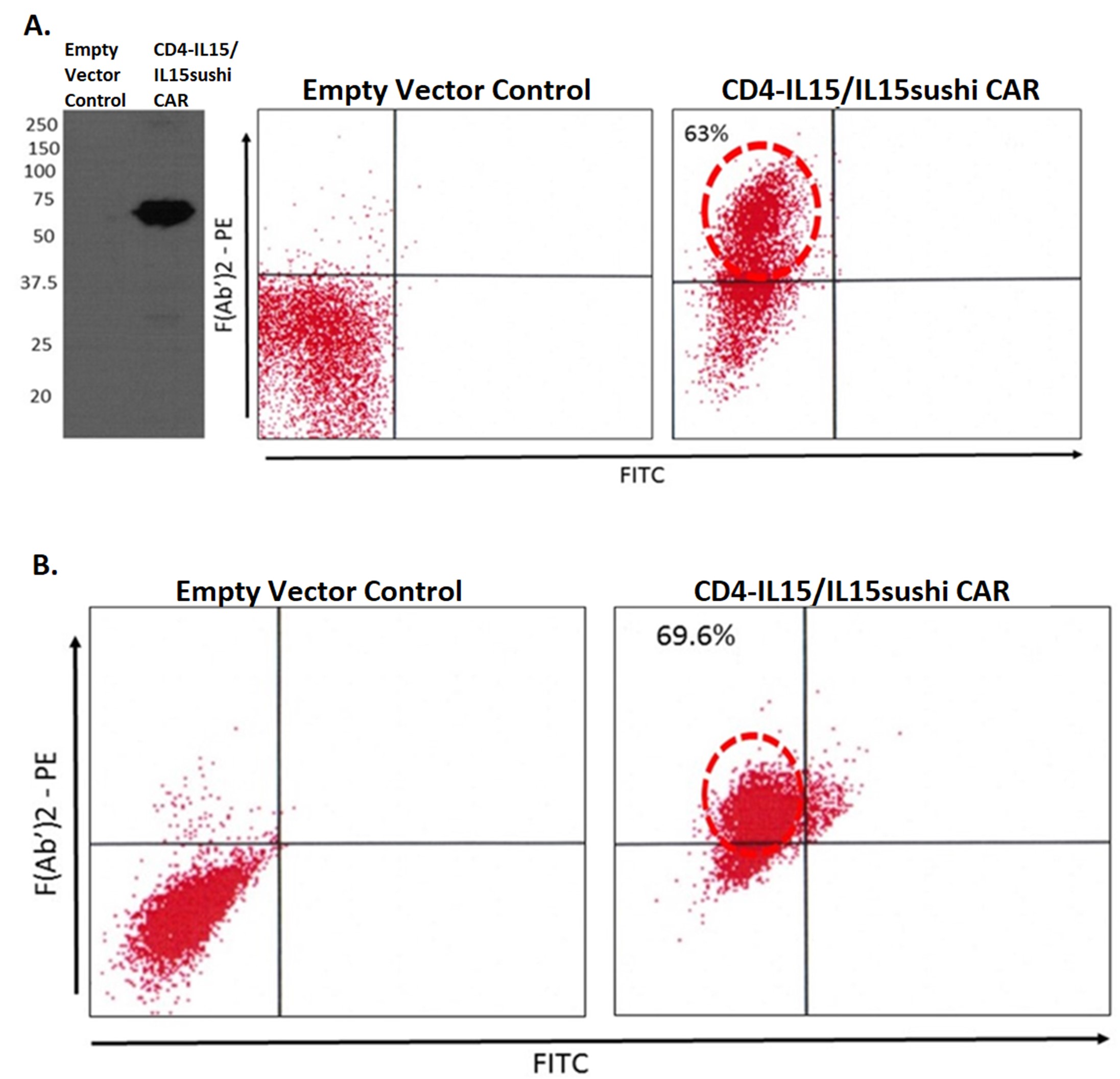

Supplement: Supplementary Figure 1 — CD4-IL15/IL15sushi Expression. (A) HEK-293FT cells were transfected with an empty vector to serve as a control (lane 1) or CD4-IL15/IL15sushi CAR (lane 2) lentiviral plasmids. Forty-eight hours after transfection, supernatant was removed, and cells were collected for Western blot analysis (left) with mouse anti-human CD3ζ antibody. Activated T cells from cord blood buffy coat were transduced with either an empty vector (control) or CD4-IL15/IL15sushi CAR viral supernatant from transfected HEK-293FT cells. Cells were stained with goat-anti-mouse F(Ab’) transduced with an empty vector control (middle) or CD4-IL15/IL15sushi CAR (right) and analyzed by flow cytometry. (B) NK92 cells were transduced similarly and analyzed by flow cytometry. [file Image_1.jpeg]

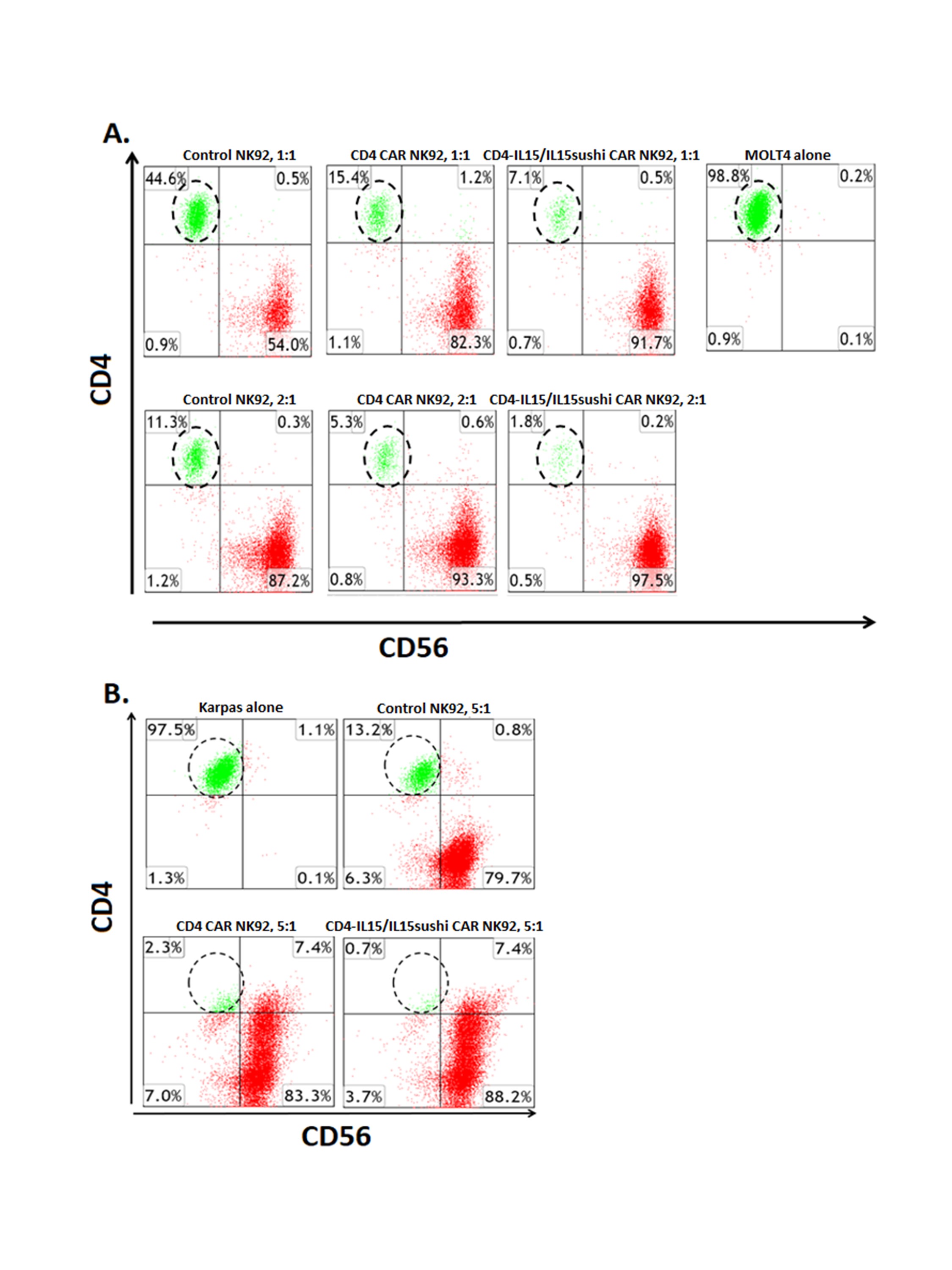

Supplement: Supplementary Figure 2 — CD4-IL15/IL15sushi CAR NK92 cells can ablate CD4+ tumors in vitro. T cells are in red. All target populations are circled and in green. (A) MOLT4 cells (right-most panel) were co-cultured with control NK92 cells, CD4 CAR NK92 cells, and CD4-IL15/IL15sushi CAR NK92 cells at an E:T ratio of 1:1 (top) and 2:1 (bottom) for 24 hours. CD4-IL15/IL15sushi CAR NK92 cells were able to lyse more target cells compared to CD4 CAR NK92 cells (84% vs. 85% at 1:1 ratio). (B) Similarly, Karpas cells (top left panel) were co-cultured with control NK92, CD4 CAR NK92, or CD4-IL15/IL15sushi CAR NK92 cells at E:T ratio of 5:1 for 24 hours. CD4-IL15/IL15sushi CAR NK92 cells again showed more potent lysis of Karpas cells compared to CD4 CAR NK92 cells (95% vs. 82%). [file Image_2.jpeg]

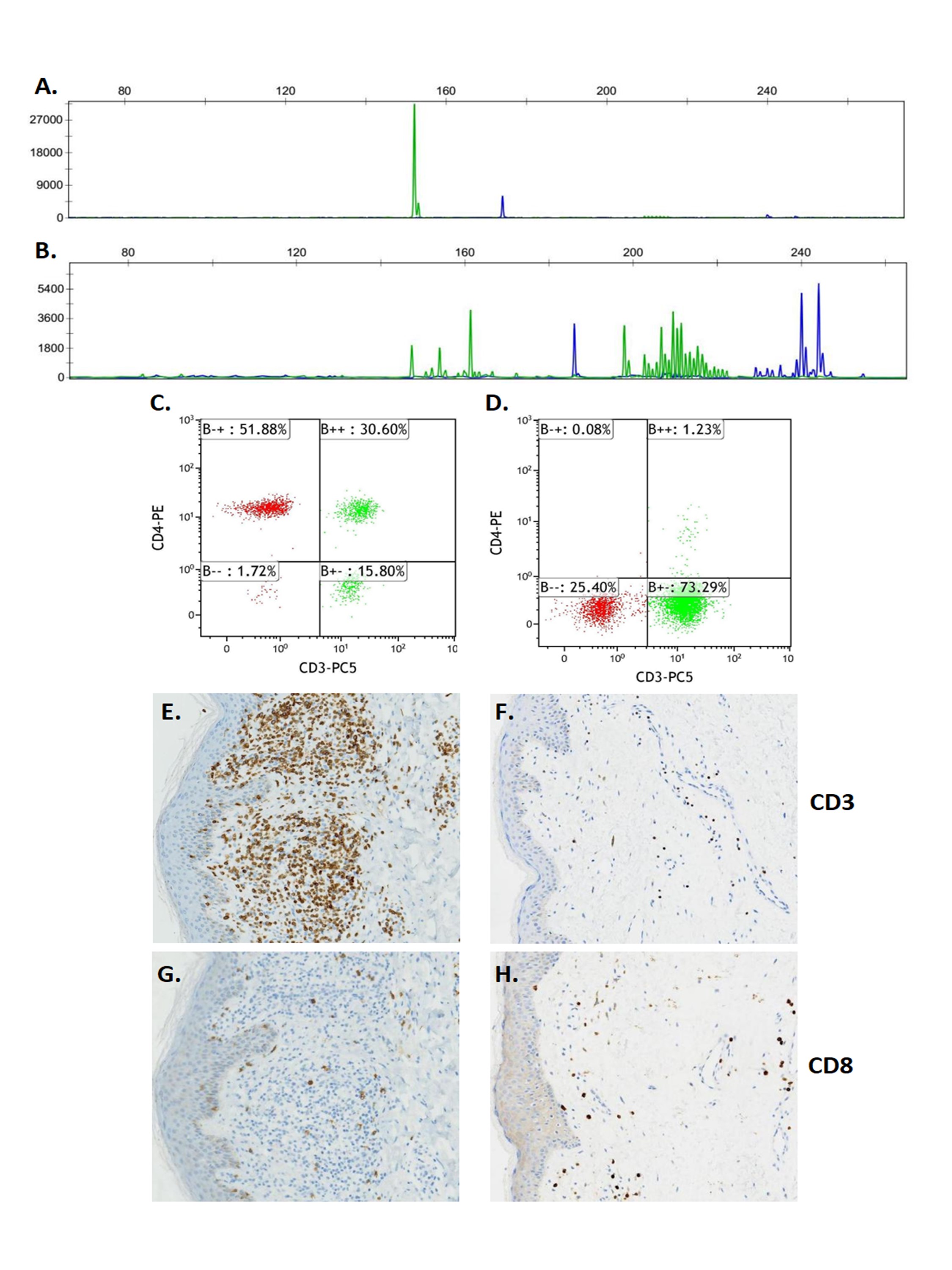

Supplement: Supplementary Figure 3 — Other findings in Patient 1 with Sézary syndrome. (A) T cell receptor (TCR) gene rearrangement before treatment with CD4-IL15/IL15sushi CAR T cells, demonstrating monoclonal rearrangement. (B) TCR rearrangement 5 months after infusion shows polyclonality with random pattern of distribution. (C) Flow cytometry results before treatment showing 51.88% CD3-CD4+ leukemia cells. (D) Flow cytometry 13 days post-therapy showing nearly undetectable levels of CD3-CD4+ leukemia cells. (E) CD3 expression in skin biopsy before treatment. CD3 expression is positive in these malignant cells as the immunohistochemistry staining detected intracellular CD3 found in the malignant cells (rather than just surface expression detected by flow cytometry). (F) CD3 expression in skin biopsy 28 days after treatment. (G) CD8 expression in skin biopsy before treatment. (H) CD8 expression in skin biopsy 28 days after treatment. [file Image_3.jpeg]

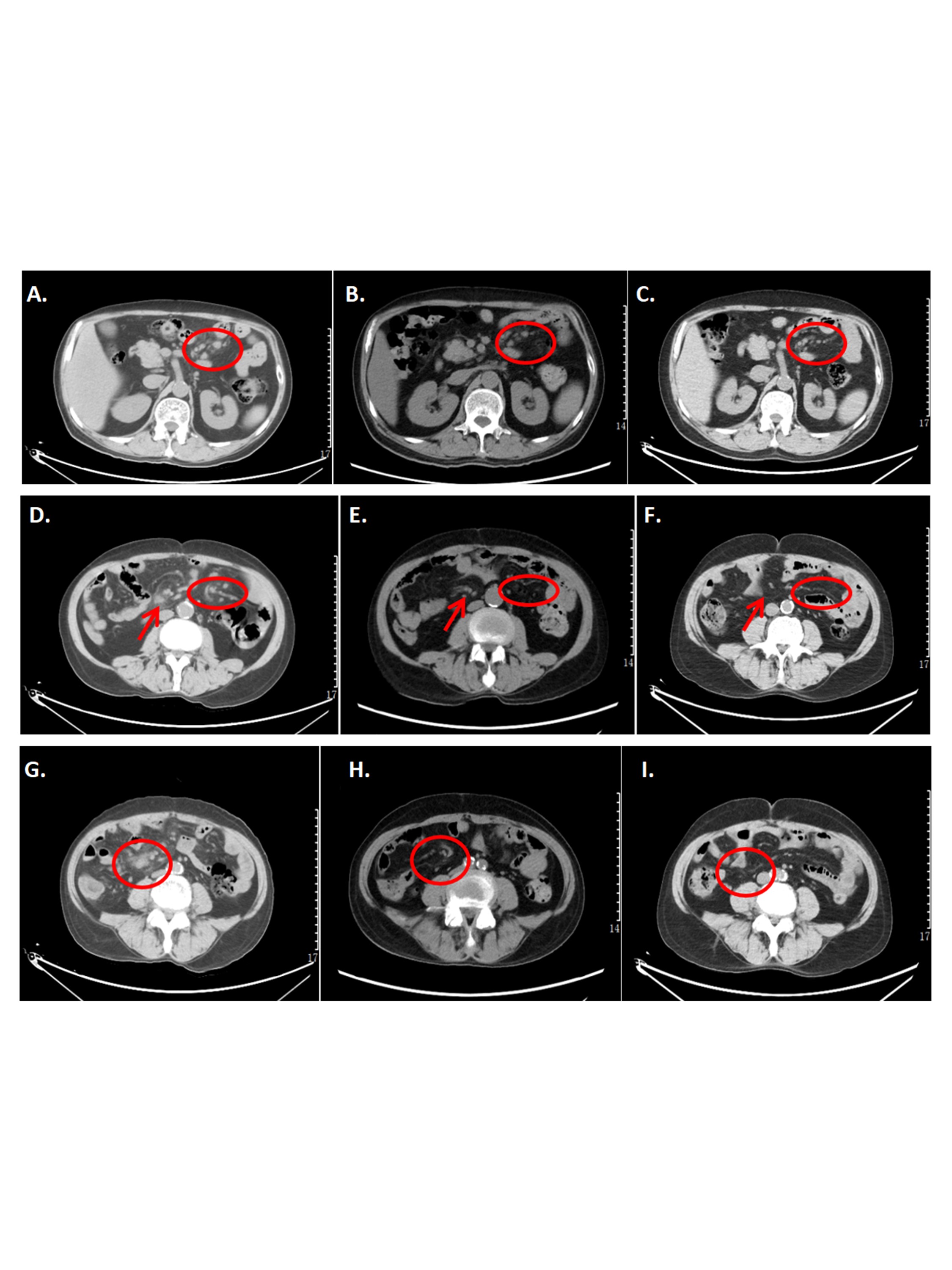

Supplement: Supplementary Figure 4 — CT scan of Patient 3 with angioimmunoblastic T cell lymphoma in mesenteric lymph nodes showing improvement after CD4-IL15/IL15sushi CAR T cell therapy. (A, D, G) CT scan before therapy. (B, E, H) CT scan on week 2. (C, F, I) CT scan on week 24 post-therapy. CT showed the gradual reduction and disappearance of multiple enlarged mesenteric lymph nodes after CD4-IL15/IL15sushi CAR T cell infusion. [file Image_4.jpeg]

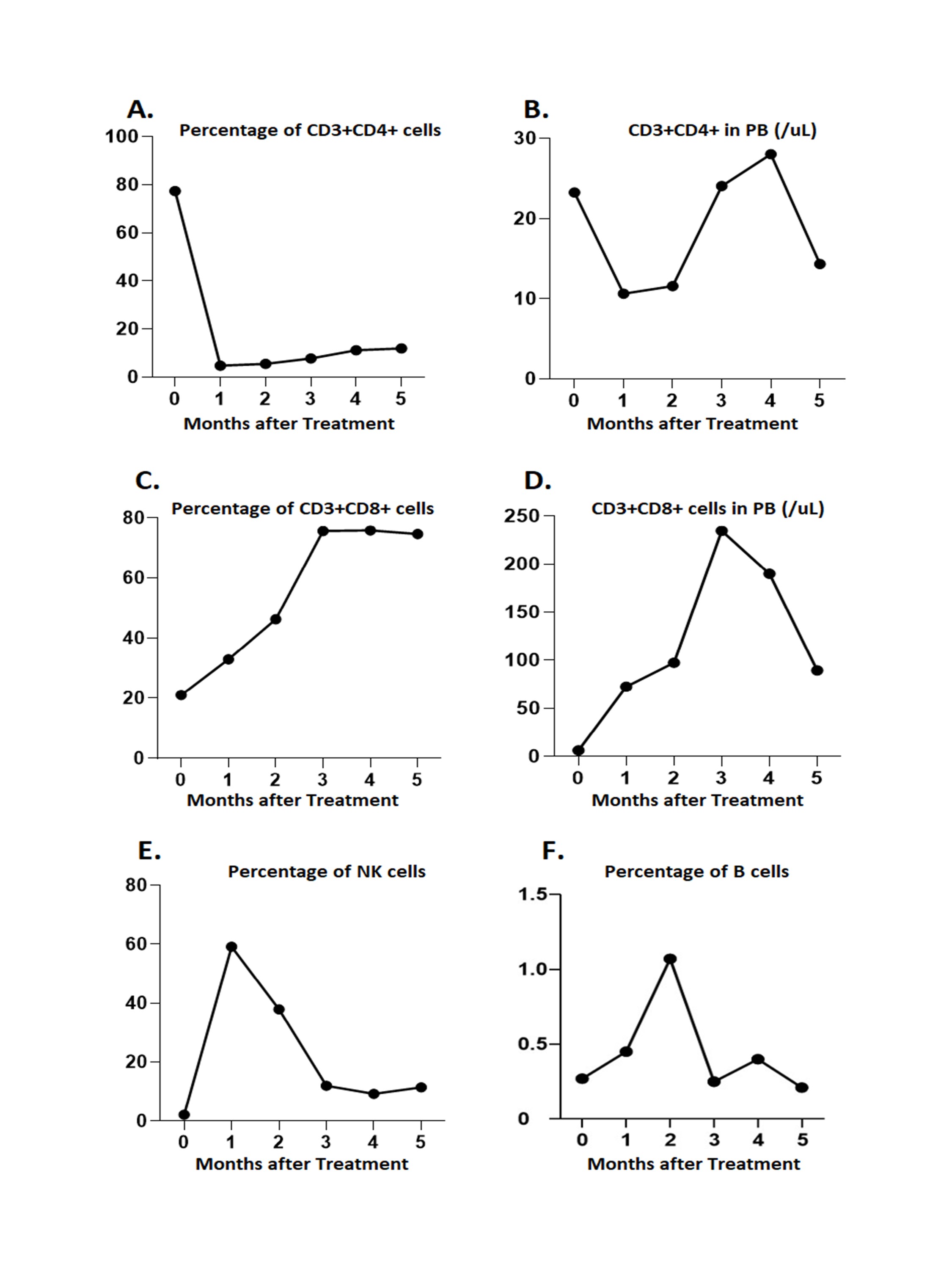

Supplement: Supplementary Figure 5 — CD4-IL15/IL15sushi CAR T cells alter the peripheral blood makeup in Patient 3 with angioimmunoblastic T cell lymphoma in mesenteric lymph nodes. (A) CD3+CD4+ cells show a marked decline after infusion of CD4-IL15/IL15sushi CAR T cells (percentage of lymphocytes). (B) The absolute number of CD3+CD4+ cells measured declined in first month post-infusion. (C) CD3+CD8+ cells showed marked expansion following therapy (percentage of lymphocytes). (D)The absolute number of CD3+CD8+ cells measured expanded during the first few months after infusion. (E) NK cells demonstrated marked expansion in the first month following treatment before returning to baseline levels. (F) B cells demonstrated variable levels following CD4-IL15/IL15sushi CAR T cell treatment. [file Image_5.jpeg]
